# miR-182 and miR-183 Promote Cell Proliferation and Invasion by Targeting FOXO1 in Mesothelioma

**DOI:** 10.3389/fonc.2018.00446

**Published:** 2018-10-22

**Authors:** Rui Suzuki, Vishwa Jeet Amatya, Kei Kushitani, Yuichiro Kai, Takahiro Kambara, Yukio Takeshima

**Affiliations:** ^1^Department of Pathology, Graduate School of Biomedical and Health Sciences, Hiroshima University, Hiroshima, Japan; ^2^Department of Surgical Oncology, Research Institute for Radiation Biology and Medicine, Hiroshima University, Hiroshima, Japan

**Keywords:** malignant mesothelioma, cell line, miR-182, miR-183, FOXO1

## Abstract

Dysregulation of miR-182 and miR-183 has been implicated in the progression of several human cancers. Our previous study showed that miR-182 and miR-183 are upregulated in malignant mesothelioma. However, their biological functions remain unclear. We performed *in-situ* hybridization to analyze the expression of miR-182 and miR-183 in human tissues. Functional analysis was performed by treatment of two mesothelioma cell lines (ACC-MESO1 and CRL-5915) with miR-182 and miR-183 inhibitors. RT-PCR and western blot analysis were conducted to analyze the expression of FOXO1, a known target of both miR-182 and miR-183. Mesothelioma cells treated with FOXO1 siRNA and miR-182/183 inhibitors were also analyzed by evaluating cell proliferation and invasion, as well as expression of FOXO1 and its downstream targets. We confirmed miR-182 expression in 25/29 cases and miR-183 expression in 29/29 cases of human mesothelioma tissue by *in-situ* hybridization. Notably, inhibition of miR-182 or miR-183 reduced cell proliferation, invasion, migration, and adhesion abilities of mesothelioma cells. Surprisingly, transfection with both miR-182 and miR-183 inhibitors showed even more effects on cell progression. Furthermore, FOXO1 was identified as a target of miR-182 and miR-183 in mesothelioma cells. Inhibition of miR-182 and miR-183 reduced cell proliferation ability via upregulation of FOXO1 and its downstream targets, namely, p27. Moreover, inhibition of miR-182 and miR-183 reduced the cell invasion properties of mesothelioma cells. Our findings indicated that miR-182 and miR-183 promote mesothelioma cell progression via downregulation of FOXO1 and p27. Targeting the miR-182/183—FOXO1 axis could serve as a novel treatment against malignant mesothelioma.

## Introduction

Malignant pleural mesothelioma is an aggressive cancer that is predominantly caused by exposure to asbestos. Although the incidence of malignant pleural mesothelioma in the United States has decreased recently, cases in Japan and Western European countries continue to increase (1), and worldwide incidence of malignant pleural mesothelioma is predicted to increase (2). In a large-scale population-based study, the 6-month, 1, and 5-year overall survivals of malignant pleural mesothelioma patients were only 55, 33, and 5%, respectively (3). Based on the findings of multiple studies, the median survival rate of Japanese patients with malignant pleural mesothelioma ranged from 2.5 to 16.3 months (4–6). The molecular mechanisms underlying the carcinogenesis of malignant mesothelioma are not yet fully understood, and effective therapeutic strategies for the treatment of this disease remain to be established.

MicroRNAs are short non-coding RNA molecules comprising 21 to 25 nucleotides that act by suppressing gene expression at the post-transcriptional level by binding to the 3′ untranslated region (3′UTR) of their target genes (7). Many microRNAs that are known as onco-microRNAs or tumor-suppressor microRNAs are associated with tumor carcinogenesis and serve as potential diagnosis markers and therapeutic targets (8–10).

In a previous study, we performed comprehensive analysis of microRNA expression patterns in mesothelioma cell lines (11). We have previously reported significant upregulation of miR-182 and miR-183 in mesothelioma cells. The cluster comprising miR-96, -182, and -183 was first reported in development of sensory organs (12). However, miR-182 expression has been implicated in the carcinogenesis of pancreatic carcinoma (13), glioblastoma (14), and lung cancer (15), while miR-183 expression has been reported to be involved in the carcinogenesis of osteosarcoma (16), glioblastoma (17), and pancreatic cancer (18).

Forkhead box O1 (FOXO1), a member of forkhead family, has been reported to regulate carcinogenesis by activating or suppressing the expression of its target genes in various human malignant tumors (19). Aberrant FOXO1 expression has been demonstrated in gastric cancer (20), lung cancer (21), osteosarcoma (22), pancreas cancer (23), and glioblastoma (24). In addition, FOXO1 has been recognized as a potential target of certain microRNAs, including miR-182 and miR-183, in endometrial cancer (25).

In the present study, we investigated the biological roles of miR-182 and miR-183 and their target, FOXO1, in mesothelioma cells.

## Materials and methods

### *In situ* hybridization of human mesothelioma tissues

Formalin-fixed and paraffin-embedded (FFPE) tissue samples collected from 30 human mesothelioma patients were retrieved from the Department of Pathology, Hiroshima University. The collection of tissue specimens for this study was carried out in accordance with the “Ethics Guidelines for Human Genome/Gene Research” enacted by the Japanese Government. Ethical approval was obtained from the institutional review committee (Hiroshima University E-974). All experimental procedures were in accordance with ethical guidelines. Samples used were linked-anonymized archival specimens and individual consent was opt-out for this research. MicroRNA expression levels were analyzed by *in situ* hybridization using Double-DIG-labeled miRCURY LNA miRNA Detection Probes and miRCURY LNA microRNA ISH Optimization Kit (FFPE) according to the manufacturer's recommended protocol with minor modifications (all purchased from Exiqon, Vedbaek, Denmark). Briefly, after de-paraffinization and incubation with protease for 10 min at room temperature, the sections were hybridized with hsa-miR-182 and hsa-miR-183 probes (40 nM) at 50°C for 2 h. The hybridized probes were detected by incubation with the anti-digoxigenin antibody (mouse monoclonal; 1:100; Santa Cruz Biotechnologies, Dallas, Texas, USA) at room temperature followed by alkaline phosphatase conjugated secondary antibody (Universal AP Multimer, Ventana/Roche Diagnostics, Tokyo, Japan) for 1 h at room temperature. Sections were visualized by treated with the AP substrate, nitro blue tetrazolium, and 5-bromo-4chloro-3-indoyl phosphate (NBT-BCIP; Roche, Tokyo, Japan) at 30°C for 30 to 60 min and subsequently counterstained with the nuclear fast red stain. Sections with U6 snRNA probe (1 nM) as the positive control and Scramble-miRNA probe (40 nM) as negative control were performed in parallel.

### Mesothelioma cell lines

The mesothelioma cell line ACC-MESO1 was purchased from RIKEN BioResearch Center, Tsukuba, Japan. The CRL-5915 cell line was obtained from the American Type Culture Collection, ATCC; Manassas, VA, USA. Mesothelioma cells were maintained in Roswell Park Memorial Institute 1640 medium with GlutaMAX and sodium pyruvate (RPMI-1640) added with 1% kanamycin/fungizone and 10% fetal bovine serum (FBS) in a humidified incubator with 5% CO_2_ at 37°C (all purchased from Gibco/Thermo Fisher Scientific, Tokyo, Japan).

### Transient transfection of mesothelioma cells with miRNA inhibitors

Mesothelioma cell lines were transfected with miRVana miRNA inhibitors, namely, miR-182 inhibitor (Anti-hsa-miR-182-5p, UUUGGCAAUGGUAGAACUCACACU), miR-183 inhibitor (Anti-hsa-miR-183-5p, UAUGGCACUGGUAGAAUUCACU), a mixture of both miR-182 and miR-183 inhibitors, or Negative Control miR-inhibitor using Lipofectamine RNAiMAX in Opti-MEM (all purchased from Thermo Fisher Scientific) according to the manufacturer's protocols.

### Co-transfection of mesothelioma cells with microRNA inhibitors and FOXO1 siRNA

Cells at 60 to 80% confluence were co-transfected with miRNA inhibitors (miR-182 inhibitor, miR-183 inhibitor, both miR-182 and miR-183 inhibitors, or negative control miRNA inhibitor) along with Silencer select siRNA (FOXO1 (assay id #s5257 and #s5258) or negative control siRNA #1 (Thermo Fisher Scientific) using Lipofectamine RNAiMAX according to the manufacturer's protocols.

### Cell proliferation assay

Mesothelioma cell lines (3 × 10^3^ cells) were incubated with 1 pmol miRNA inhibitor or miRNA with 5 pmol siRNA in Opti-MEM in 96-well plates in triplicate for 3 days. Cell proliferation rates (based on ATP activity, an indicator of metabolically active cells) were determined at 24, 48, and 72 h using Cell Titer-Glo 2.0 reagent (Promega KK, Tokyo, Japan) on a GloMax Explorer microplate reader (Promega) according to the manufacturer's recommended protocols.

### Cell invasion assay

ACC-MESO1 (1 × 10^5^ cells) and CRL-5915 (3 × 10^5^ cells) were incubated with 5 pmol miRNA inhibitor or miRNA with 5 pmol siRNA in BD FluoroBlok culture inserts with 8-μm pores (BD Biosciences, Franklin Lakes, NJ, USA) and coated with Geltrex Matrigel (Thermo Fisher Scientific) according to the manufacturer's protocols. Considering that ACC-MESO1 cells were more invasive than CRL-5915 cells, ACC-MESO1, and CRL-5915 cells were analyzed at 24 and 48 h after transfection, respectively. Invasive cells were stained with Hoechst 33342 (Thermo Fisher Scientific) for 10 min, and the images of the invasive cells were acquired using a fluorescence microscope with DAPI filter. The total numbers of invading cells were determined by analyzing the fluorescence images using CellProfiler cell imaging software (26).

### Cell migration assay

Cell migration was analyzed by conducting a wound/scratch assay. Mesothelioma cell lines were incubated overnight with 5 pmol miRNA inhibitors in collagen-coated 24-well plates. Wounds were created by scratching the wells with 1,000 μl micropipette tips. The wells were washed twice to remove floating cells and subsequently incubated with fresh RPMI-1640 containing 5% FBS. Microscopic images were acquired at 0, 12, and 24 h (ACC-MESO1) or 0, 24, and 48 h (CRL-5915). The area of wound in percentage was determined using Scratch software (27).

### Cell adhesion assay

Mesothelioma cell lines (ACC-MESO1 and CRL-5915) were incubated with 5 pmol microRNA inhibitors for 6 h in 24-well plates, and floating cells were removed by washing twice with fresh growth medium. The number of adherent cells was determined using Cell Titer-Glo 2.0 reagent (Promega) according to the manufacturer's recommended protocols on a GloMax Explorer microplate reader (Promega).

### Real-time reverse transcription polymerase chain reaction (RT-qPCR)

Mesothelioma cells were transfected with 25 pmol miRNA inhibitors and siRNAs in six-well plates for 24 to 72 h. RNA was extracted from cells transfected with the microRNA inhibitors and siRNA transfected using a Maxwell RSC simplyRNA Cells kit (Promega) on a Maxwell® RSC Instrument (Promega) according to the manufacturer's protocol. A total of 200 ng of RNA was reverse-transcribed with SuperScript IV VILO Master Mix (Thermo Fisher Scientific) and amplified with PowerUp SYBR Green Master Mix (Thermo Fisher Scientific) on a Mx3000P real-time PCR system (Stratagene, La Jolla, CA, USA). Relative expression levels were calculated following the comparative CT (ΔΔCT) method. Expression levels were normalized against GAPDH expression levels. Primer sequences used for RT-qPCR are shown in Table 1.

**Table 1:** RT-PCR primer sequences.

**Gene**	**Forward**	**Reverse**
*GAPDH*	ACAACTTTGGTATCGTGGAAGG	GCCATCACGCCACAGTTTC
*FOXO1*	TCGTCATAATCTGTCCCTACACA	CGGCTTCGGCTCTTAGCAAA
*p21*	TGTCCGTCAGAACCCATGC	AAAGTCGAAGTTCCATCGCTC
*p27*	TAATTGGGGCTCCGGCTAACT	TGCAGGTCGCTTCCTTATTCC

### Western blot analysis

Cells (5 × 10^5^ cells) were seeded and transfected in six-well plates overnight. Cell lysates were obtained from cells transfected with microRNA inhibitor and siRNA inhibitor using Cell Ly Ex (TOYO B-Net, Tokyo, Japan). Total proteins (20–30 μg) were electrophoresed on SureCast Acrylamide Gel (Thermo Fisher Scientific) at 180 V for 50 min and subsequently transferred onto polyvinylidene difluoride (PVDF) membranes using Mini Bolt Module (Thermo Fisher Scientific) at 20 V for 60 min. After blocking with PVDF Blocking Reagent for Can Get Signal (TOYOBO, Osaka, Japan), membranes were incubated overnight with primary antibodies [anti-FOXO1 antibody (1:1000, rabbit monoclonal, 2880; CST), anti-p21 antibody (1:1,000, rabbit monoclonal, 2947; CST), anti-p27 antibody (1:1,000, rabbit monoclonal, 3686; CST), and anti-GAPDH antibody (1:1,000, rabbit monoclonal, 2118; CST)] in Can Get Signal Solution 1 (TOYOBO). Afterwards, membranes were incubated with the secondary antibody [rabbit (1:1,000, anti-rabbit IgG, HRP-linked antibody 7074S; CST)]. The membranes were stained with ImmunoStar LD (Wako Pure Chemical Industries, Osaka, Japan). Band intensities were measured using a c-Digit Blot Scanner (LICOR, Lincoln, NE, USA).

### Statistical analysis

Data are expressed as mean ± standard deviation (*SD*) from three independent experiments. For statistical analysis, we used one-way ANOVA to compare subgroups. Significant difference was defined as *p*-value < 0.05.

## Results

### miR-182 and miR-183 are expressed in human mesothelioma tissues

**Figure 1:**
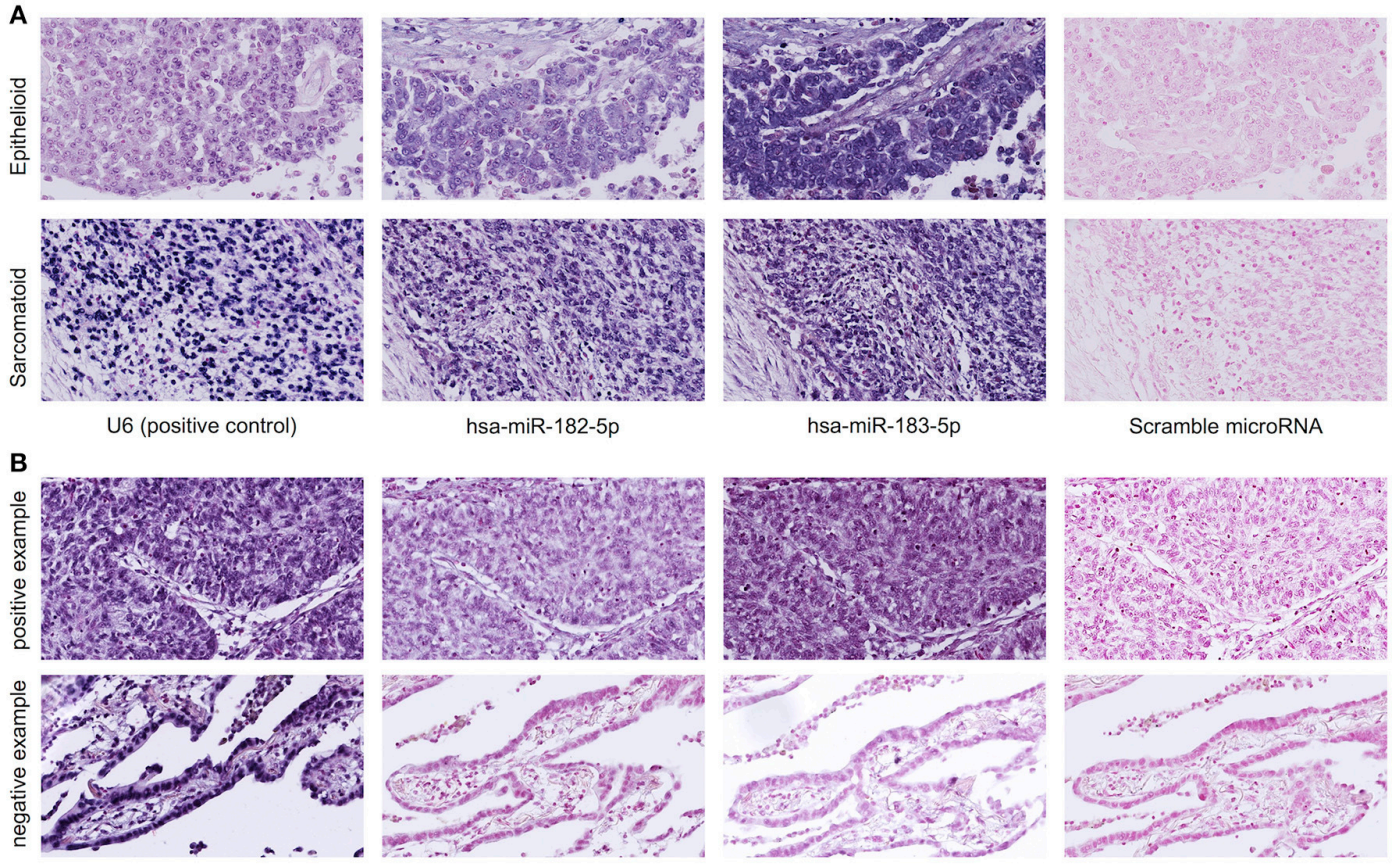
**(A)** MicroRNA *in situ* hybridization of human mesothelioma tissues shows miR-182 and miR-183 expression in the cytoplasm and nuclei of mesothelioma cells. U6 probe is the positive control, and scramble probe is the negative control. Upper panel shows representative images of epithelioid mesothelioma. Lower panel shows representative images of sarcomatoid mesothelioma. **(B)** The specificity of miR-182 and miR-183 probes is confirmed by *in situ* hybridization of lung cancer tissues showing both expression of miR-182 and miR-183 in lung squamous cell carcinoma (upper panel and no expression in lung adenocarcinoma (lower panel).

*in-situ* miRNA hybridization showed miR-182 expression in 21 of 24 epithelioid mesothelioma cases and from 4 of 5 sarcomatoid mesothelioma cases and miR-183 expression in all of 24 cases of epithelioid mesothelioma and in all of 5 cases of sarcomatoid mesothelioma (Figure 1).

### Inhibition of miR-182 and miR-183 reduces cell proliferation, invasion, migration, and adhesion

**Figure 2:**
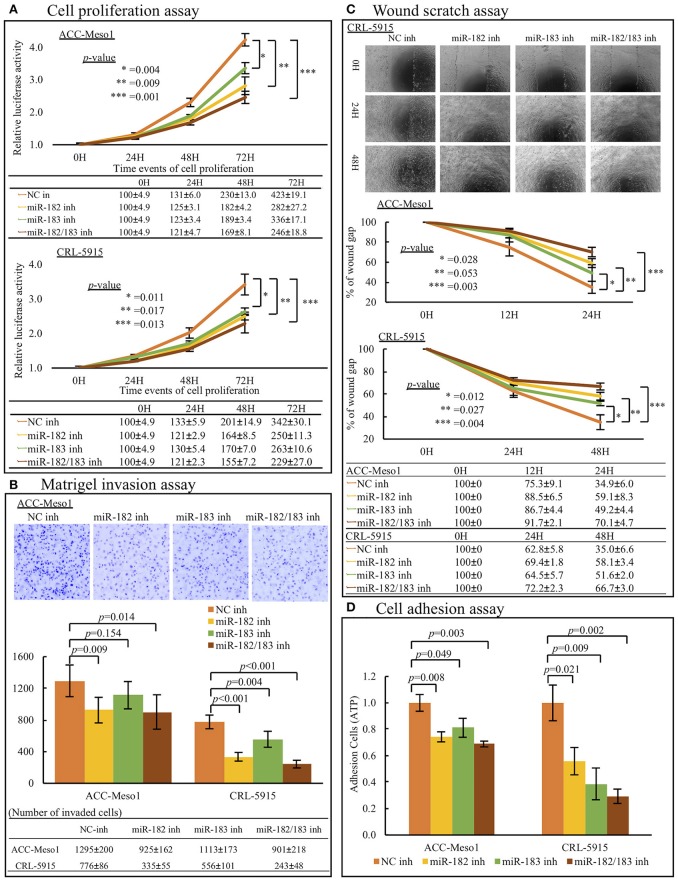
**(A)** Cell proliferation analysis. Mesothelioma cell lines (ACC-MESO1 and CRL5915) transfected with miR-182, miR-183, and a combination of both inhibitors (miR-182 inh, miR-183 inh, miR-182/183 inh) for 1, 2, and 3 days showed increasingly reduced cell proliferation compared to cells treated with negative control miRNA inhibitor (NC inh). Data were acquired on a microplate reader using Cell Titer Glo reagent (*p* < 0.05). **(B)** Invasion assay. Mesothelioma cell lines transfected with miR-182 inhibitor, miR-183 inhibitor, and a combination of both inhibitors for 24 (ACC-MESO1) or 48 (CRL-5915) h showed reduced invasiveness through the Matrigel compared to cells treated with negative control miRNA inhibitor (*p* < 0.05 except between NC-inhibited and miR-183-inhibited ACC-MESO1 cells). Upper panel figures show representative inverted fluorescence images acquired using CellProfiler Image analysis software. **(C)** Migration assay. Mesothelioma cells (ACC-MESO1 and CRL-5915) transfected with miR-182 inhibitor, miR-183 inhibitor, and a combination of both inhibitors showed reduced migration abilities compared to cells transfected with negative control miRNA mimic (*p* < 0.05 except between NC-inhibited and miR-183-inhibited ACC-MESO1 cells). Upper panel figures are representative images acquired using an inverted phase contrast microscope. **(D)** Cell Adhesion assay. Mesothelioma cells (ACC-MESO1 and CRL-5915) transfected with miR-182 inhibitor, miR-183 inhibitor, and a combination of both inhibitors showed reduced adhesion compared to cells transfected with negative control miRNA inhibitor (*p* < 0.05).

Mesothelioma cells transfected with miR-182 or miR-183 inhibitors showed reduced cell proliferation rates compared to cells treated with negative control inhibitor. Moreover, cells co-transfected with miR-182 and miR-183 inhibitors showed the lowest proliferation rates in 4 subgroups (Negative Control, miR-182 inhibitor, miR-183 inhibitor, miR-182/183 inhibitor) (Figure 2A).

In invasion assay, inhibition of miR-182 significantly reduced cell invasion. Although ACC-Meso1 transfected with miR-183 inhibitor did not show significant difference, CRL-5915 transfected with miR-183 inhibitor showed lower invasion compared to cells transfected with negative control inhibitor. Furthermore, cells co-transfected with both miR-182 and miR-183 inhibitor showed the least invasion (Figure 2B).

Moreover, mesothelioma cells transfected with miR-182 or miR-183 inhibitor showed lower cell migration abilities than cells treated with negative control inhibitor. Cells transfected with both inhibitors showed the lowest cell migration abilities (Figure 2C).

Furthermore, treatment with miR-182 and/or miR-183 inhibitors reduced the adherent ability of mesothelioma cells (Figure 2D). This result indicated that miR-182 and miR-183 may help mesothelioma cells metastasize by promoting adhesion of cells to other places.

### FOXO1 is a target of miR-182 and miR-183

We investigated the targets of miR-182 and miR-183 to further understand their biological roles in mesothelioma. Analysis using TarBase v7.0, an online database of the experimentally verified microRNA targets (http://www.microrna.gr/tarbase), showed that FOXO1 is a validated target of both miR-182 and miR-183 (28).

To verify whether FOXO1 is a target of miR-182 and miR-183 in mesothelioma cells, we conducted RT-PCR and western blot analysis of cells treated with miRNA inhibitors.

**Figure 3:**
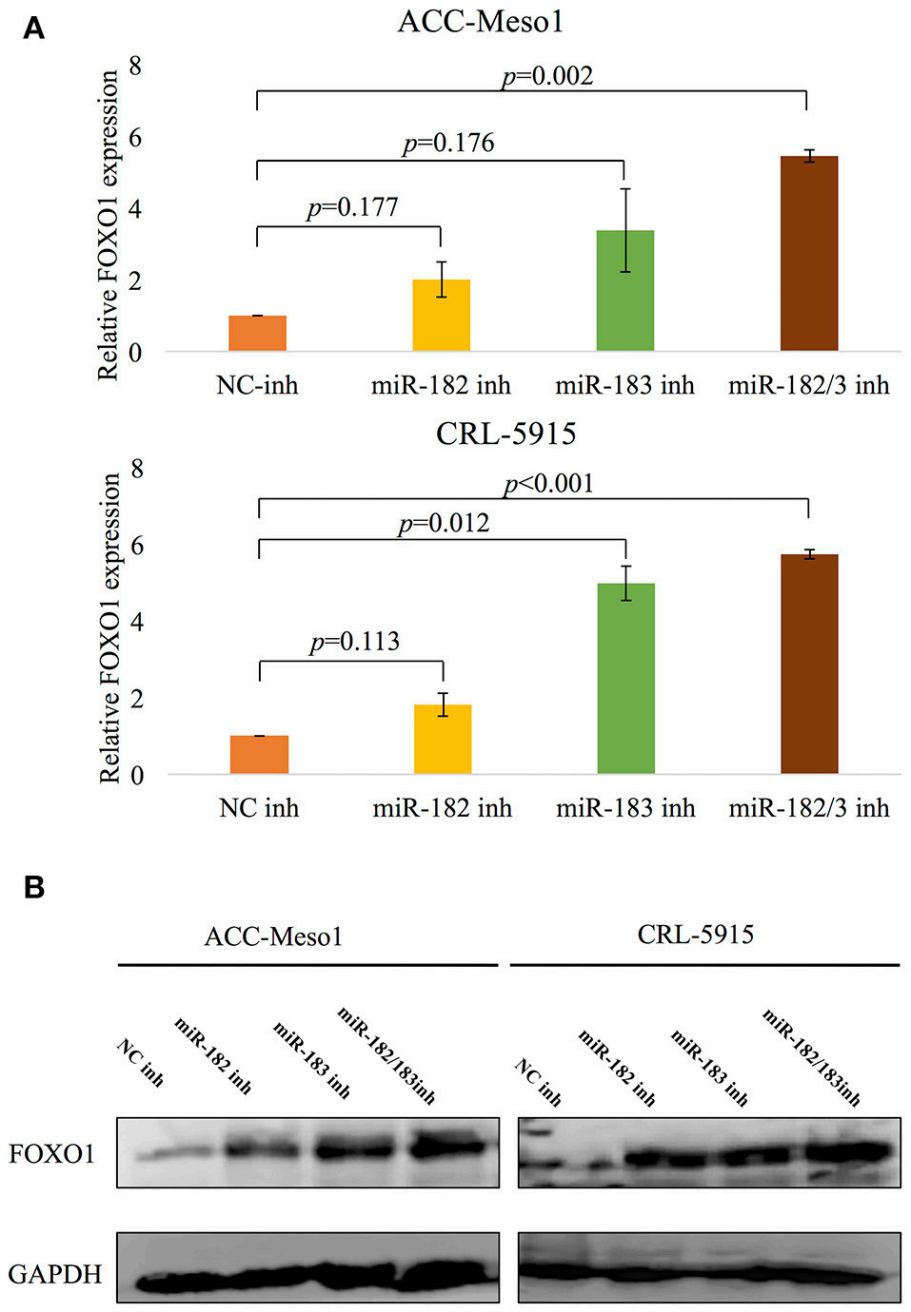
**(A)** RT-PCR analysis shows upregulation of FOXO1 mRNA expression following treatment with miR-182 or miR-183 inhibitor. Inhibition of both miR-182 and miR-183 lead to even more upregulation of FOXO1. **(B)** Results of western blot analysis showing upregulation of FOXO1 protein expression following treatment with inhibitors of miR-182, miR-183, or a combination of both miRNAs.

Mesothelioma cells treated with miR-182 and miR-183 inhibitors showed upregulated mRNA (Figure 3A) and protein (Figure 3B) expression of FOXO1. Furthermore, cells treated with both miR-182 and miR-183 inhibitors showed significant upregulation of FOXO1 expression than cells treated with miR-182 or miR-183 inhibitor alone.

### FOXO1 downregulation is important for miR-182- and miR-183-induced cell proliferation and invasion in mesothelioma

**Figure 4:**
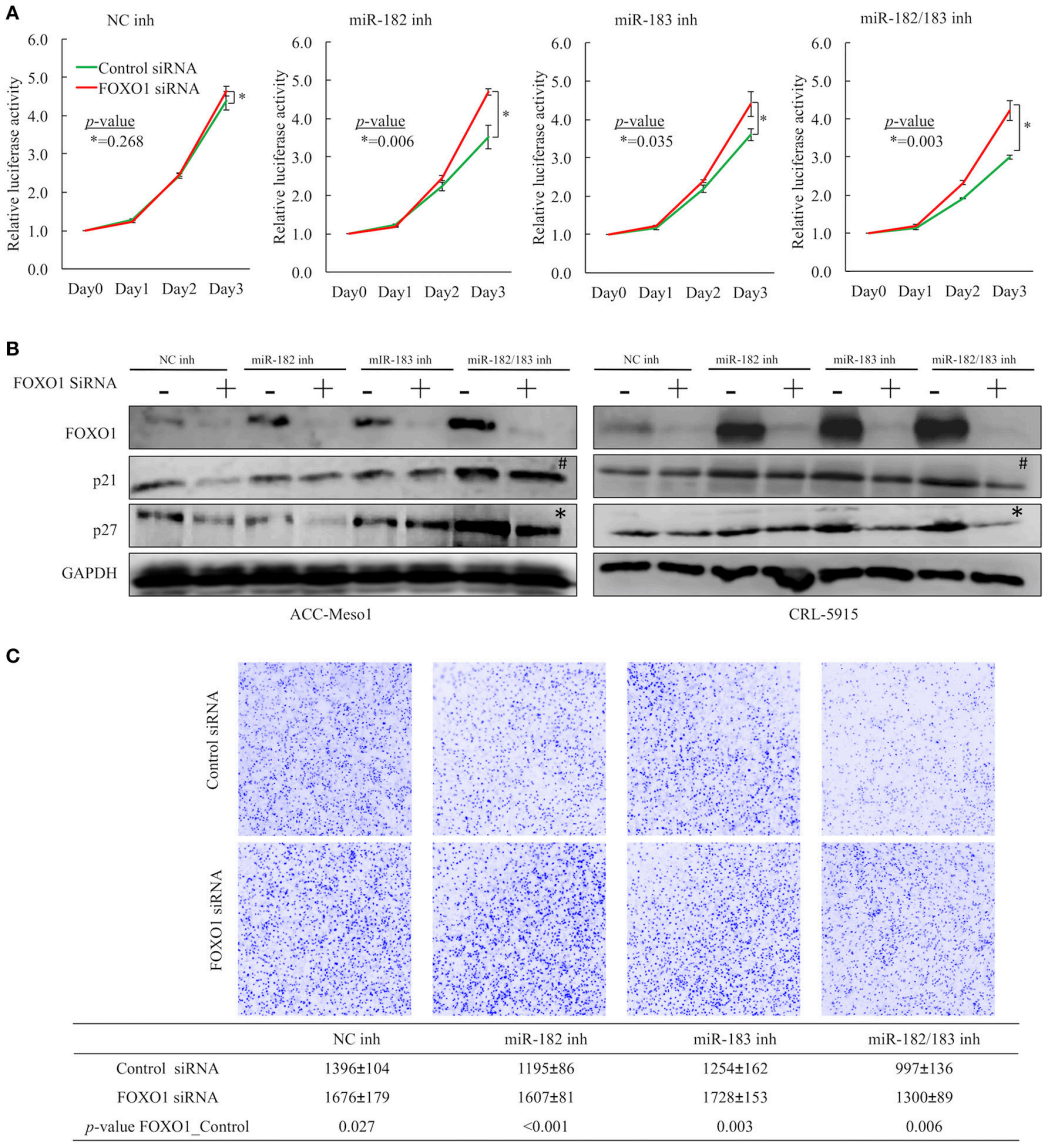
**(A)** Results of cell proliferation assay showing that transfection with FOXO1 siRNA counteracted the growth-suppression effects of miR-182 and miR-183 inhibitors when cells were co-transfected with microRNA inhibitors and FOXO1 siRNA. **(B)** Western blot showing FOXO1 siRNA countered the upregulated expression of FOXO1 by miRNA inhibitors. Moreover, FOXO1 siRNA also downregulated p27 expression in cells treated with both miR-182 and miR-183 inhibitors (^*^), but not clear with p21 expression (#). **(C)** Results of invasion assay demonstrating that transfection with FOXO1 siRNA counteracted the cell invasion-promoting effects of miR-182 and miR-183 inhibitors when cells were co-transfected with microRNA inhibitors and FOXO1 siRNA.

To determine whether miR-182 and miR-183 inhibit mesothelioma cell proliferation and invasion by suppressing FOXO1 expression, we conducted cell proliferation and invasion assays by co-transfecting ACC-MESO1 and CRL-5915 cells with microRNA inhibitors and siRNAs. Cell proliferation and invasion were not significantly reduced in cells transfected with FOXO1 siRNA despite the observed miRNA inhibition upon treatment with miR-182 and miR-183 inhibitors when compared to mesothelioma cells transfected with negative control siRNA (Figures 4A,C). To further investigate the miR-182/183-FOXO1 pathway, we evaluated the mRNA and protein expression levels of FOXO1 in cells transfected with negative control siRNA. The transfection of mesothelioma cells with miR-182 and miR-183 inhibitors also upregulated the protein expression levels of p21 and p27. However, FOXO1 knockdown inhibited the upregulation of p27 expression but not p21 expression (Figure 4B). In addition, FOXO1 knockdown reversed the cell invasion-reducing effects of miR-182 and miR-183 inhibitors (Figure 4C).

## Discussion

Malignant pleural mesothelioma is fatal cancer that is mostly caused by asbestos exposure. Malignant pleural mesothelioma has poor prognosis and is associated with survival periods ranging from 5 to 13.2 months (29).

Pemetrexed and platinum combination chemotherapy is the standard and first line of treatment for inoperable malignant mesothelioma. Recently, the combination of a conventional anticancer drug (cisplatin/pemetrexed) and Bevacizumab, a recombinant monoclonal antibody for the human vascular endothelial growth factor, has been reported to prolong patient survival (30). However, the applicability of this treatment for mesothelioma remains to be established. Therefore, there is an urgent need to develop novel therapeutic regimes for advanced mesothelioma, which can facilitate the management of the predicted peak incidence of malignant mesothelioma.

The present study focused on microRNAs as novel therapeutic targets for the treatment of malignant mesothelioma. MicroRNAs are short, non-coding RNAs that regulate gene expression, and various microRNAs are known to regulate tumor progression. In fact, certain miRNAs have entered the preclinical and clinical stages as therapeutic targets, and these recently developed treatments are expected to be available in the market. Anti-miR-10b has been investigated as a therapeutic target for glioblastoma and is currently at the preclinical stage. The use of a miR-34 mimic for the treatment of liver cancer is currently undergoing phase 1 clinical trials. The use of anti-miR-122 has been studied for its protective effects against HCV infection and is currently undergoing phase 2a clinical trials (31).

Birnie et al. reviewed the role of microRNAs in malignant mesothelioma. MicroRNAs, such as miR-31, miR-let-7a/b, and miR-34a, have been reported as potential therapeutic targets for the treatment of malignant mesothelioma. MiR-126 and miR-21 have been recognized as potential diagnostic markers, and miR-let-7c-5p and miR-151a-5p have been identified as potential prognostic markers for mesothelioma (32). In a previous study, we performed comprehensive analysis of microRNA expression in mesothelioma cell lines. In particular, we identified miRNAs that are downregulated in mesothelioma, such as miR-1 and miR-214, and upregulated microRNAs, such as miR-182 and miR-183. The tumor-suppressing microRNAs, namely, miR-1 and miR-214, play roles in promoting mesothelioma cell proliferation by targeting PIM1 (11). In the present study, we identified the novel roles of miR-182 and miR-183 as onco-microRNAs in mesothelioma cell lines. We showed the association of miR-182 and miR-183 with proliferations of mesothelioma cells. We also found the association of miR-182 and miR-183 with invasion, migration, and adhesion of mesothelioma cells by conventional assays. However, it needs to be clarified whether invasion, migration, and adhesion are influenced by the proliferation due to miRNAs of mesothelioma cells by more precise experiments like real-time cell tracking analysis.

**Figure 5:**
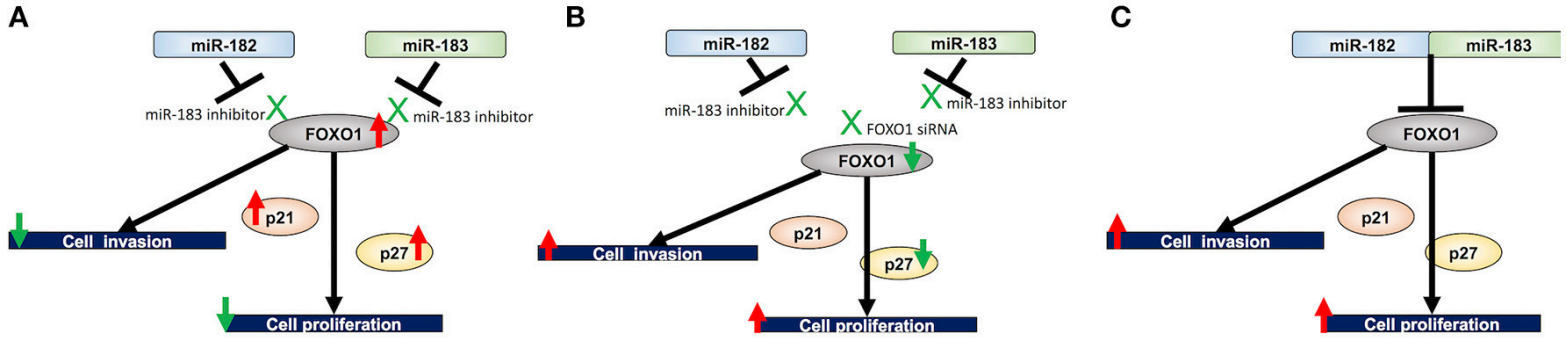
Proposed miRNA-182/183 FOXO1 p21/27 signaling pathway. **(A)** Inhibition of miR-182 and miR-183 upregulates the expression of FOXO1, which in turn inhibits cell proliferation probably by acting on its downstream targets, namely, p21 and p27, and invasive ability of mesothelioma cells. **(B)** Treatment with FOXO1 siRNA counteracts the inhibitory effects of miR-182 and miR-183. **(C)** Proposed mechanisms underlying the effects of miR-182 and miR-183 on cell proliferation and invasion assay through FOXO1.

Furthermore, we investigated the experimentally validated targets of miR-182 and miR-183 in mesothelioma cells. FOXO1 is an experimentally validated target of miR-182 in breast cancer (28) and a target of miR-183 in non-small cell lung cancer (33). Furthermore, FOXO1 is an experimentally validated target of both miR-182 and miR-183 in T helper 17 cells (34). Our current findings identified FOXO1 as a target of both miR-182 and miR-183 in mesothelioma cells. FOXO1 is a member of forkhead box O (FoxO) family and was discovered in Drosophila as a member of the homeotic gene family (35), which is highly conserved throughout evolution, with four main members in mammals, namely, FOXO1, FOXO3, FOXO4, and FOXO6 (36). The phosphatidylinositol-3-kinase (PI3K) pathway is known to play an important role in many biological functions, including cell proliferation (37). In this pathway, AKT as the upstream regulators of FOXO family prevents FOXO1 from transferring to nuclei by its phosphorylation and thereby regulates FOXO1 activity (38). FOXO1 is a transcription factor that regulates various biological events, including gluconeogenesis, differentiation, cell growth, and apoptosis (19). FOXO1 downregulation is known to be involved in cancer progression in some human malignancies. FOXO1 knockdown has been demonstrated to promote prostate tumorigenesis and cell invasion properties (39). In the present study, we analyzed the role of FOXO1 by conducting cell proliferation and invasion assays using FOXO1-siRNA in mesothelioma cells treated with miR-182 and miR-183 inhibitors and demonstrated the association between FOXO1 and mesothelioma cell proliferation and invasion. Furthermore, we investigated the expression of the primary downstream targets of FOXO1, namely, p21 and p27. Results showed that p21 and p27 expression levels were upregulated in mesothelioma cells treated with miR-182 and miR-183 inhibitors, and the observed upregulation of p27 was reversed by FOXO1 knockdown but not p21. Based on the above-mentioned findings, we hypothesized that miR-182 and miR-183 can suppress the expression of FOXO1, p21, and p27. Therefore, our findings indicated that miR-182/183 acts through the FOXO1-p27 axis in proliferation of mesothelioma cells (Figure 5). The suppression of p21 expression in mesothelioma cells by miR-182/183 needs further clarification.

In conclusion, we demonstrated that miR-182 and miR-183 promote cell proliferation and invasion by targeting FOXO1 in mesothelioma cells. In addition, our findings demonstrated the role of the miR-182/183-FOXO1-p27 axis in promoting cell proliferation in mesothelioma cells. Therefore, miR-182 and miR-183 and FOXO1 can serve as potential therapeutic targets for treatment of malignant mesothelioma.

## Author contributions

RS, VA, and YT designed the study. VA and YT supervised and facilitated the study. RS, KK, YK, and TK performed the experiments. RS analyzed the data. RS and VA interpreted the results and wrote the manuscript.

### Conflict of interest statement

The authors declare that the research was conducted in the absence of any commercial or financial relationships that could be construed as a potential conflict of interest.
